# In Vitro Antimicrobial Activity and Probiotic Potential of *Bifidobacterium* and *Lactobacillus* against Species of *Clostridium*

**DOI:** 10.3390/nu11020448

**Published:** 2019-02-21

**Authors:** Cinara R. A. V. Monteiro, Monique S. do Carmo, Bruna O. Melo, Matheus S. Alves, Camilla I. dos Santos, Sílvio G. Monteiro, Maria Rosa Q. Bomfim, Elizabeth S. Fernandes, Valério Monteiro-Neto

**Affiliations:** 1 Programa de Pós-graduação em Ciências da Saúde, Universidade Federal do Maranhão, Av. dos Portugueses, campus do Bacanga, São Luís 65065545, MA, Brazil; cinaraaragao@hotmail.com (C.R.A.V.M.); carmo.monique@outlook.com (M.S.d.C.); 2 Programa de Pós-graduação, Universidade Ceuma, Rua dos Castanheiros No. 1, jardim Renascença II, São Luís 65075120, MA, Brazil; brunaoliv.96@gmail.com (B.O.M.); matheusjoc@hotmail.com (M.S.A.); camilla_itapary@hotmail.com (C.I.d.S.); silvio.monteiro@ceuma.br (S.G.M.); mrqbomfim@yahoo.com.br (M.R.Q.B.); elizabeth.soares@ceuma.br (E.S.F.); 3 Departamento de Biologia, Universidade Federal do Maranhão, Av. dos Portugueses, campus do Bacanga, São Luís 65065545, MA, Brazil

**Keywords:** probiotics, *Lactobacillus plantarum*, *Clostridium*, dysbiosis, antimicrobial activity

## Abstract

Many *Clostridium* species are found as commensal members of the intestinal microbiota. However, imbalances of the microbiota may lead to certain infections caused by these microorganisms, mainly *Clostridium butyricum*, *Clostridium difficile*, and *Clostridium perfringens*. In many cases, infection recurrence can occur after antibiotics, indicating the need for novel therapeutic options that act on the pathogens and also restore the microbiota. Herein, the in vitro antimicrobial activity and probiotic potential of clinical and reference strains of *Bifidobacterium* and *Lactobacillus* were investigated against *Clostridium* species. Antimicrobial activity was evaluated by the agar spot test and inhibition of gas production. Then, the probiotic potential of selected strains was assessed by analyzing their coaggregation ability, adhesive properties to host cells and mucin, tolerance to acidic pH and bile salts, and antimicrobial susceptibility profiles. *Lactobacillus plantarum* ATCC 8014 was the most promising strain based on its inhibitory activity against *Clostridium* spp. Also, this strain met criteria to be considered a probiotic based on its coaggregation ability, adhesive properties, and tolerance to harsh pH and bile acid salt conditions. The results indicate that among the studied strains, *L. plantarum* ATCC 8014 presents probiotic potential for controlling infections induced by the studied *Clostridium* species and should be further evaluated in in vivo animal models.

## 1. Introduction

In humans, the normal intestinal microbiota consists of a large number and high diversity of commensal microorganisms (about 10^13^ to 10^14^), mainly in the large intestine, where they establish a symbiotic relationship that influences the entire host organism [1,2,3]. Intestinal homeostasis is maintained through complex interactions between the host’s immune system and the microbiota [4,5]. However, this mutualistic relationship can be disrupted by a variety of factors, such as changes in diet, use of antibiotics, and immunomodulatory drugs, among others, leading to changes in both bacterial function and diversity [6,7].

An imbalance in the intestinal microbiota may cause or contribute to the establishment of infectious and inflammatory diseases such as inflammatory bowel disease [8], antibiotic-associated diarrhea (AAD) [9], irritable bowel syndrome [10], and necrotizing enterocolitis (NEC) [11]. In addition, there is growing evidence in the literature on the association of dysbiosis with other non-infectious diseases, including type 2 diabetes [12], asthma [13], non-alcoholic fatty liver disease [14], colorectal cancer [15], neurological conditions [16], and cardiovascular disease [17].

In some infectious disorders of the intestinal tract resulting from microbiota disruption, certain specific bacteria have been implicated as etiologic agents, especially potentially pathogenic *Clostridium* species. For example, *Clostridium difficile* is described as one of the leading causes of diarrhea and colitis associated with antibiotic use, with detection frequency ranging from 13% to 28% [9,18]. Mortality rates in patients with *C. difficile* infection may exceed 30%, especially in those individuals experiencing recurrence of infection within six months of initial treatment [19].

NEC is the most common and serious intestinal disorder among preterm infants and is diagnosed through radiological findings that include, among other manifestations, the presence of intestinal pneumatosis [20]. Its incidence may reach 12% in children that weigh less than 1 kg at birth [21] and mortality may range from 20% to 50% [22]. Risk factors for NEC include those that affect the normal microbiota, such as neonatal immaturity, enteral feeding, and intestinal colonization [23]. Its etiology is controversial and several causative organisms have been proposed, including viruses, *Staphylococcus* spp., various gram-negative bacilli, and *Clostridium* spp. [24]. Recent studies point to *Clostridium butyricum* as an important cause of NEC [25,26,27], although there are non-toxigenic strains that can be employed as probiotics [28].

*Clostridium perfringens* causes infection in both humans and animals [29]. Depending on the toxigenic type, *C. perfringens* may also cause other disorders in the intestinal tract, such as food poisoning and necrotic enteritis, in addition to tissue infections accompanied by myonecrosis, such as gas gangrene due to trauma [29]. In humans, *C. perfringens* has also been reported in cases of AAD with a lower prevalence [9,30].

Antibiotic therapy is the first line of treatment for these infectious intestinal disorders. However, recurrence is frequent, particularly in cases of AAD, since the microbiota remains unbalanced due to the use of broad-spectrum antibiotics [9,19]. Alternative interventions have been employed, such as narrow-spectrum antibiotics, dietary changes, fecal transplantation, and probiotics, which may attenuate the clinical symptoms, restore diversity of the intestinal microbiota, and improve host health [31,32,33].

Some studies have shown alleviation of AAD [34,35] and NEC [36,37] with probiotics. The efficacy of distinct probiotic strains, especially among *Bifidobacterium* and *Lactobacillus* species, suggests that they may have common properties that could positively impact patient health in such pathological states [31,38]. However, the basis of these properties is not yet fully understood [39] and there are no compelling explanations for the effects of probiotics in AAD or NEC. Several plausible mechanisms have been investigated and may contribute to the observed health benefits [39,40], but in terms of translational research, this is an evident shortcoming that hinders the development of improved therapies. 

Therefore, the objective of this study was to carry out an in vitro screening of clinical and reference strains of *Bifidobacterium* and *Lactobacillus* with antimicrobial activity against *C. butyricum*, *C. difficile*, and *C. perfringens*. The most promising strain was subjected to analysis of criteria for consideration as a potential probiotic, including its ability to adhere to eukaryotic cells and mucus and its tolerance to acidic pH and bile salts.

## 2. Materials and Methods

### 2.1. Bacterial Strains and Growth Conditions

The following *Bifidobacterium* and *Lactobacillus* reference strains were studied: *Bifidobacterium longum* subsp. *longum* ATCC 15707, *Lactobacillus brevis* ATCC 367, *Lactobacillus delbrueckii* subsp. *delbrueckii* ATCC 9649, *Lactobacillus fermentum* ATCC 23271, *Lactobacillus paracasei* subsp. paracasei ATCC 335, *Lactobacillus plantarum* ATCC 8014, and *Lactobacillus rhamnosus* ATCC 9595, which were obtained from the National Institute of Quality Control in Health (INCQS, FIOCRUZ, Rio de Janeiro, Brazil). *Lactobacillus rhamnosus* GG (LGG, ATCC 53103) was isolated from a commercial probiotic product (Floridral—Pharmaforce ApS, Copenhagen, Denmark) and used as a positive control. In addition, fecal isolates from newborn infants were evaluated and maintained in the Culture Collection Sector of Ceuma University, including *Bifidobacterium longum* 49.3, *Bifidobacterium animalis* subsp. *lactis* 56.1, *Bifidobacterium bifidum* 14.2, and *Lactobacillus fermentum* 54.2. All *Bifidobacterium* and *Lactobacillus* isolates were routinely cultured on agar or MRS broth (Man-Rogosa-Sharpe, Difco-BD, Detroit, MI, USA) with 0.25% l-cysteine and incubated at 37 °C for 24–48 h under anaerobic conditions. Cultures were stored in MRS broth with 20% glycerol at −80 °C.

*C. butyricum* ATCC 860, *C. difficile* ATCC 9689, and *C. perfringens* ATCC 12924 were obtained from INCQS (FIOCRUZ, Rio de Janeiro, Brazil). *Clostridium* strains were cultured in RCM (reinforced clostridial medium, Acumedia, Lansing, MI, USA) or thioglycolate medium (Acumedia) and incubated at 37 °C for 24–48 h in an anaerobic atmosphere. They were stored in RCM with 20% glycerol at −80 °C. 

### 2.2. Antimicrobial Activity Screening

The ability of potential probiotics to inhibit *Clostridium* growth was evaluated in two distinct assays. All assays were performed in triplicate over three days.

#### 2.2.1. Agar Spot Test

The agar spot test procedure was performed as described previously [41], with modifications. Briefly, in a Petri dish containing 10 mL of MRS agar, 5 μL of each probiotic culture was spotted onto one quadrant of the culture medium, followed by incubation at 37 °C for 24 h under anaerobic conditions. After incubation, 10 mL of thioglycolate agar was overlaid onto the MRS agar containing the growth of *Bifidobacterium* and *Lactobacillus* strains. After solidification of the culture medium at room temperature (25–28 °C), *Clostridium* spp. suspensions (McFarland standard No. 0.5, 1.5 × 10^8^ colony-forming units per milliliter (CFU/mL)) were spread with the aid of a swab. The plates were incubated at 37 °C for 24 h under anaerobic conditions. The formation of a clear halo around growth of the probiotics was indicative of antimicrobial activity. The diameter of the growth inhibition halo was measured and expressed in millimeters.

#### 2.2.2. Inhibition of Gas Production

The ability of probiotics to inhibit the growth of *Clostridium* strains was also evaluated by assessing the inhibition of gas production due to the fermentative action of the pathogens, as described previously [42], with some modifications. Briefly, the assay was performed by inoculating 1 μL (~10^7^ UFC) of *Clostridium* culture into the upper third of the RCM agar layer (supplemented with 1.5 g/100 mL bacteriological agar), composed of 3 mL per tube. Subsequently, 3 mL of MRS containing 0.7 g% bacteriological agar was melted, cooled to 50 °C, and inoculated with 30 μL (~10^8^ UFC) of each probiotic culture. The contents were homogenized by vortexing and immediately poured over the RCM agar layer in tubes inoculated with the *Clostridium* strains. RCM agar with *Clostridium* and MRS agar without inoculated probiotics were used as negative controls. The tubes were incubated under anaerobic conditions at 37 °C for 24 h. The assays were performed in triplicate, with and without buffering of the MRS medium with K_2_HPO_4_ and KH_2_PO_4_ (100 mM). A positive assay for antimicrobial activity was characterized by the absence of gas production, that is, in the absence of bubbles in the culture media, or medium breakage.

### 2.3. Antimicrobial Activity after pH Adjustment

The strain with the highest antimicrobial activity was selected to verify if growth inhibition of *Clostridium* spp. was due to the acidic pH. Culture supernatants were tested after pH adjustment essentially as described by Gaspar et al. [43]. After cultivation of the microorganism in MRS broth (Difco-BD), 10 mL of the bacterial culture was heated at 70 °C for 30 min to inhibit protease activity, cooled at room temperature, and centrifuged (5,000 × *g* for 15 min at 4 °C). Hydrogen peroxide was eliminated by the addition of 5 mg/mL catalase from bovine liver (Sigma-Aldrich, St Louis, MO, USA) followed by filtration through a 0.2 μm pore-size cellulose acetate (Whatman^®^, Clifton, NJ, USA). Antimicrobial activity of the cell-free culture supernatants (CFCN) was evaluated with and without pH adjustment to 6.5 with 10 M NaOH solution. Antimicrobial activity was evaluated by the antagonist well-diffusion method. Briefly, 20 mL of MRS soft agar (0.7% agar) was inoculated with 200 μL of each *Clostridium* strains. Wells with 4 mm diameter were punched in agar plates and filled with 100 μL of CFCN. Phosphate-buffered saline (PBS, pH 7.4) was used as a negative control. Plates were incubated under anaerobic conditions at 37 °C for 24 °C in an upright position. The inhibition zone diameters were measured and expressed in millimeters.

### 2.4. Coaggregation Test

After cultivation of the probiotic strains and *Clostridium* species, aliquots of 1 mL of each culture were washed twice with phosphate-buffered saline (PBS, pH 7.2), centrifuged at 5000× *g* for 15 min, and resuspended in PBS. The optical density of each suspension was adjusted (OD_620nm_ = 0.1), and 500-μL aliquots of the probiotic suspensions were mixed with 500 μL of each pathogen suspension in 24-well plates (Nunc, Roskilde, Denmark) and incubated at 37 °C for 4 h under constant stirring (100 rpm) on an orbital shaker. Plates were observed for macroscopically visible clumps and under inverted microscopy [44]. Glass slides were also prepared with 5 μL of each suspension and evaluated under the microscope for visualization of bacterial coaggregates after Gram staining. *L. fermentum* ATCC 23271 was used as a positive control in this assay, since high coaggregation scores were previously demonstrated [45]. Control assays were performed with individual bacterial samples to assess their ability to autoaggregate.

### 2.5. Mucin Binding Assay

The ability of selected probiotic strains to bind to mucin was evaluated essentially as described by Tallon et al. [46]. A volume of 100 μL of a 10 mg/mL mucin solution in PBS (pH 7.2) was added to the wells of polystyrene microtiter plates (Nunc) and incubated overnight at 4 °C. The wells were washed twice with 200 μL PBS and saturated with a 2% (w/v) bovine serum albumin (BSA) solution (Sigma-Aldrich, St. Louis, MO, USA) for 4 h at 4 °C. Finally, the wells were washed twice with 200 μL PBS. At least four replicates were used to estimate the adhesion of a given strain. Probiotic cultures in MRS broth were washed three times in PBS, and the final suspension was standardized by spectrophotometry (OD_600nm_ = 0.1). Aliquots of 100 μL of the bacterial suspension were added to each well, and the microplates were incubated at 37 °C for 1 h. After this, the wells were washed 12 times with 1 mL PBS to remove non-adherent bacteria. The wells were treated with 200 μL of 0.5% Triton X-100 (Sigma-Aldrich), and the plates were then incubated for 2 h at room temperature under orbital shaking to release the adhered bacteria. Then, the wells were scraped with a sterile tip and the number of bacteria with binding ability to mucin was estimated by serial decimal dilutions in PBS and plating on MRS agar, followed by incubation at 37 °C for 24 h under anaerobic conditions. *L. fermentum* ATCC 23271 was used as a positive control [45]. Mucin-containing wells without bacteria were used as negative controls. 

### 2.6. Adhesion to Eukaryotic Cells

Adhesion to HeLa was evaluated according to the method of Carmo et al. [45]. A 300-μL aliquot of each potential probiotic cultured in MRS broth was washed three times with PBS (pH 7.4, Sigma-Aldrich) and the bacterial pellet was resuspended in 300 μL Dulbecco’s modified Eagle’s medium (DMEM, Sigma-Aldrich). Monolayers of HeLa cells grown in 24-well microplates (Nunc) containing DMEM supplemented with 10% fetal bovine serum (Gibco, Gaithersburg, MD, USA), with or without glass coverslips, were inoculated with 50 μL (~2.3 × 10^7^ CFU) of bacterial suspension and incubated at 37 °C under 5% CO_2_ for 3 h. Then, each well was washed three times with PBS to remove non-adherent bacteria. For quantification of the adherent bacteria, the HeLa cell monolayers in the wells without coverslips were treated with 1 mL of 0.1% Triton X-100 (Sigma-Aldrich) for 5 min and scraped with the aid of a tip. Thereafter, serial decimal dilutions were spread on MRS agar plates and incubated at 37 °C for 24 h. The total number of bacteria adhered to the cells was expressed as CFU per milliliter. Wells with HeLa cells incubated in the absence of bacteria were used as negative controls. Visualization of bacterial adherence to eukaryotic cells was performed after fixation with methanol (Amresco, Gymea, Australia) and staining with May–Grunwald and Giemsa (Amresco). Gram staining was also used to better visualize gram-positive *Lactobacillus* adhered to the cells. The coverslips were then mounted on glass slides and visualized by light microscopy under a 100× oil immersion objective.

### 2.7. Tolerance to Acidic pH and Bile Salts

Tolerance of selected bacteria to acidic pH (2 and 4) and bile salts (0.5% and 1%, Oxgall, Sigma-Aldrich) was evaluated as previously described, with minor modifications [47]. Briefly, 900 μL MRS, adjusted to pH 2 or 4, or non-adjusted (control), or supplemented with 0%, 0.5%, or 1.0% (*w*/*v*) Oxgall (Sigma-Aldrich), was inoculated with 100 μL of a 24-h culture, which had been previously washed three times with PBS and resuspended in the same volume of MRS broth. After incubation at 37 °C for 3 h under anaerobic conditions, the percentage of viable bacteria relative to that in the control was determined by plate counting on MRS agar.

### 2.8. Antibiotic Susceptibility Testing

Antibiotic susceptibility of probiotics was determined by a modification of the agar overlay diffusion method, as previously described [48]. Commercial discs (Oxoid) containing different antibiotics, including ciprofloxacin (5 μg), clindamycin (2 μg), chloramphenicol (30 μg), erythromycin (15 μg), gentamicin (10 μg), penicillin (10 μg), rifampicin (5 μg), co-trimoxazole (25 μg), vancomycin (30 μg), and tetracycline (30 μg), were placed on MRS agar plates inoculated with *Lactobacillus* (10^8^ CFU/mL). The plates were incubated anaerobically at 37 °C for 24 h. Antibiotic susceptibility was evaluated based on the diameter (in millimeters) of the growth inhibition zone around the discs [48]. The reference strain *Staphylococcus aureus* ATCC 25923 was used for quality control of antibiotic discs and tested in Mueller–Hinton agar, as recommended [49].

### 2.9. Statistical Analyses

Statistical analyses were performed using NCSS 11 Statistical Software (2016; NCSS, Kaysville, UT, USA). Adherence to eukaryotic cells and to mucin was expressed as log CFU/mL (±SD). Tolerance to acidic pH and to bile salts was compared to growth in standard MRS medium. The Shapiro–Wilk test was carried out and confirmed that all variables were normally distributed. Thus, all comparisons were carried out by the Student *t*-test. Statistical significance was established at *p* < 0.05. All assays were performed in three independent experiments conducted on three different days.

## 3. Results

### 3.1. Selection of Strains with Antimicrobial Activity against Clostridium spp.

The antimicrobial effects of potential probiotics, as determined by the agar spot test, are shown in Table 1. *C. butyricum* was inhibited by 11 strains (91.7%), whereas *C. difficile* and *C. perfringens* were inhibited by 9 (75%) strains each. The diameter of the inhibition zone varied among the clinical and reference probiotic strains. Of the 12 species tested, 8 (66.7%) exhibited antimicrobial activity against all three *Clostridium* species. The largest inhibition zones were produced by *L. plantarum* ATCC 8014, mainly against *C. butyricum* (17 mm). Only *B. animalis* 56.1 showed no inhibitory activity against any *Clostridium* species in this assay.

**Figure 1:**
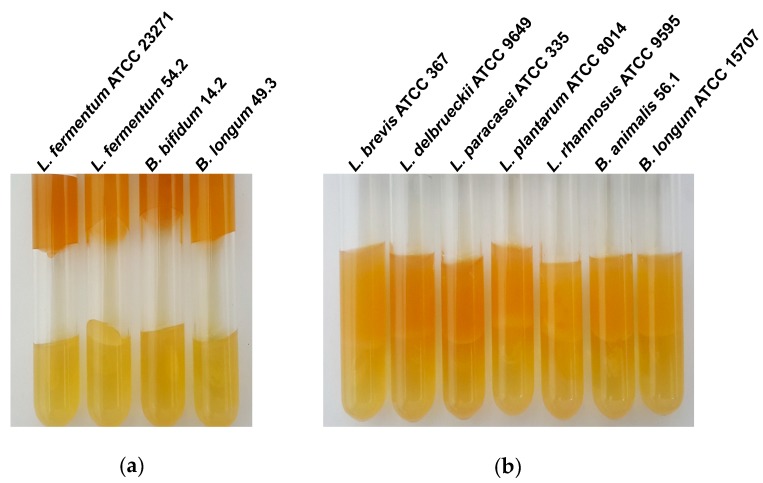
Inhibition of gas production assay. The lower layer corresponds to the RCM agar inoculated with *Clostridium butyricum* ATCC 860 and the upper layer is MRS medium with 0.7% agar and 100 mM phosphate buffer inoculated with *Bifidobacterium* and *Lactobacillus* strains; cultures were incubated at 37 °C for 24 h under anaerobic conditions. (**a**) Gas production by *C. butyricum* in buffered MRS medium, indicating the absence of inhibitory activity of *Lactobacillus* and *Bifidobacterium* strains. (**b**) Inhibitory activity of five *Lactobacillus* and two *Bifidobacterium* strains on gas production by *Clostridium butyricum*.

To assess whether or not the inhibitory activity on *Clostridium* spp. was due to acid produced by the probiotics, a preliminary attempt was made by supplementing MRS with phosphate buffer to evaluate the inhibition of gas production by *C. butyricum* ATCC 860 in culture medium. This test was also carried out in MRS medium without phosphate buffer. *C. butyricum* ATCC 860 was selected because this strain usually produces a large amount of gas resulting from its fermentative activity in thioglycolate medium or RCM. In the presence of buffer, the strains *L. brevis* ATCC 367, *L. delbrueckii* ATCC 9649, *L. paracasei* ATCC 335, *L. plantarum* ATCC 8014, *L. rhamnosus* ATCC 9595, *B. animalis* 56.1, and *B. longum* ATCC 15707 inhibited gas production by *Clostridium* spp., whereas the other strains did not (Figure 1). Among the strains that inhibited gas production in the absence of the phosphate buffer, only *B. bifidum* 14.2 gave a negative result when the assay was carried out in the of presence phosphate buffer in the MRS medium (Table 2).

*L. plantarum* ATCC 8014, the strain with the highest antimicrobial activity in the agar spot test for the three *Clostridium* strains, was selected for investigation of the antimicrobial activity in cell-free culture supernatants (CFCN) with and without pH adjustment to 6.5 with NaOH 10N, after growth in MRS broth. CFCN at pH 4.3, the final pH after cultivation of *L. plantarum* ATCC 8014, showed growth inhibition zones ranging from 14.2 (±0.8) mm to 16.5 (±0.8) mm, whereas CFCN at pH 6.5 presented inhibition zones from 13.2 mm (±0.4) mm to 14.7 (±0.5) mm. However, the inhibition zones of the CFSN at pH 4.3 in comparison to CFSN at pH 6.5 only presented statistical significance against *C. butyricum* (*p* < 0.05, Table 3).

### 3.2. L. plantarum ATCC 8014 Presents Probiotic Potential

**Figure 2:**
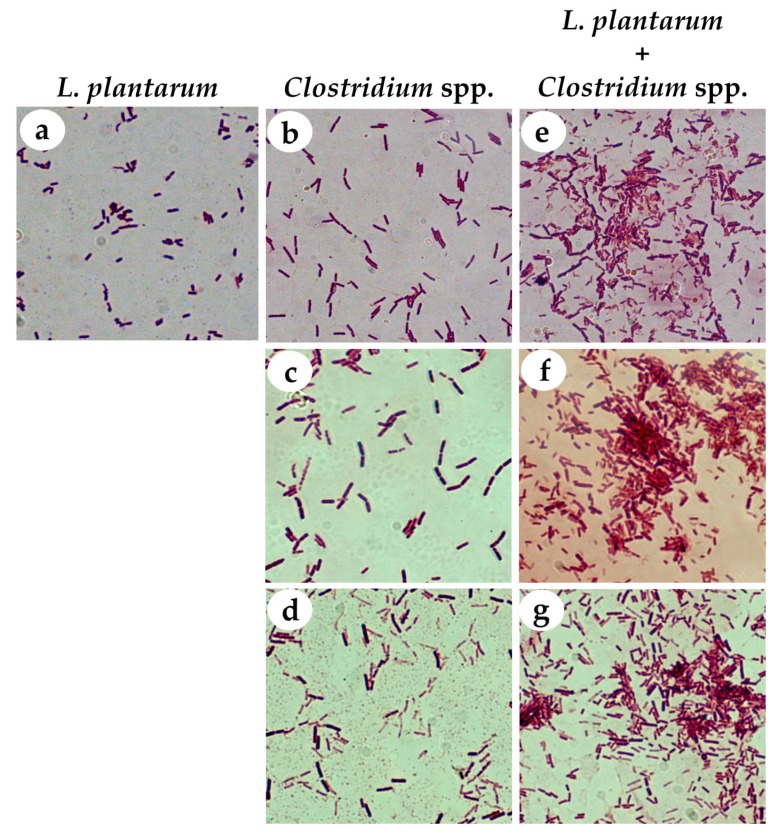
Gram staining of bacterial isolates before (**a**–**d**) and after (**e**–**g**) the coaggregation assays of *L. plantarum* ATCC 8014 with the three species of *Clostridium.* (**a**) *L. plantarum*, (**b**) *C. butyricum*, (**c**) *C. difficile,* and (**d**) *C. perfringens* controls. Coaggregation of *L. plantarum* and (**e**) *C. butyricum*, (**f**) *C. difficile,* or (**g**) *C. perfringens*. Formation of bacterial aggregates were observed after mixing *L. plantarum* with each *Clostridium* strain. Gram stained slides were visualized by light microscopy under a 100× oil immersion objective.

*L. plantarum* ATCC 8014 was selected for further analysis and characterization as a potential probiotic. In terms of its coaggregation capacity, we observed that *L. plantarum* ATCC 8014 interacted clearly with the pathogens, forming bacterial aggregates with the three species of *Clostridium* (Figure 2).

**Figure 3:**
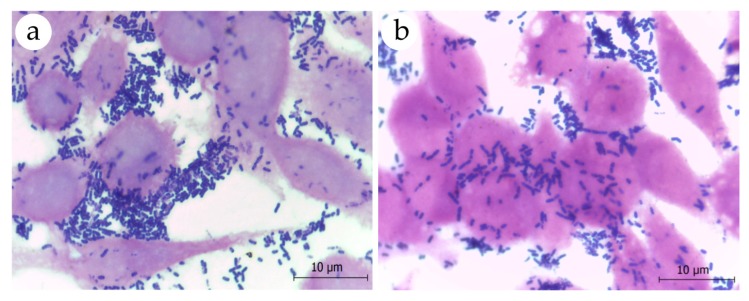
Microscopic visualization of adhesion assays of (**a**) *L. plantarum* ATCC 8014 and (**b**) *L. fermentum* ATCC 23271 to HeLa cells after inoculation of approximately 10^7^ CFU of bacterial suspensions. Cell monolayers grown on coverslips were stained by the Gram’s method and examined by light microscopy under a 100× oil immersion objective. Note the presence of numerous gram-positive bacilli adhered to HeLa cells.

Investigation of the adhesion properties of *L. plantarum* ATCC 8014 revealed its ability to interact with eukaryotic cells, as observed in the assay with HeLa cells (Figure 3). In fact, adherence values for *L. plantarum* ATCC 8014 were higher than those observed for *L. fermentum* ATCC 23271 (Table 4, *p* = 0.0037). In contrast, *L. fermentum* ATCC 23271 exhibited a greater ability to bind mucin than *L. plantarum* ATCC 8014 (Table 4, *p* < 0.0001).

*L. plantarum* ATCC 8014 exhibited growth at pH 2 and 4 after 180 min of exposure, with growth values of 70.3% and 97.8%, respectively, relative to that observed in standard MRS medium (Table 4). Assays performed at bile salt concentrations of 0.5% and 1.0% resulted in growth values of 110.8% and 90.1%, respectively, relative to that observed in standard medium. No differences were observed relative to the growth of *L. fermentum* strain ATCC 23271 (Table 5).

The antibiotic susceptibility profile of *L. plantarum* ATCC 8014 was evaluated using the overlay diffusion method. *L. plantarum* ATCC 8014 showed resistance to ciprofloxacin and vancomycin and sensitivity to all other antibiotics tested (Table 6).

## 4. Discussion

Some probiotic strains have been successfully used in clinical studies for the treatment or prevention of AAD [34,35] and NEC [36,37]. Their mechanisms of action still remain unclear, perhaps because it seems that this is not important since some probiotics interventions have been effective in certain patients with some reliability [40,50]. On the other hand, controversial findings regarding AAD and NEC interventions have been reported in the literature, as a positive effect does not always occur when used in cases of dysbiosis [51]. A better understanding of the relevant “central probiotic properties” that contribute to their inhibitory effects on major etiological agents of these clinical syndromes could therefore aid in the design of a more rational strategy for selecting and producing more effective probiotics [31].

In this study, we showed that different clinical and reference strains of *Bifidobacterium* and *Lactobacillus* present different levels of antimicrobial efficacy against *C. butyricum*, *C. difficile*, and *C. perfringens*. Evaluation of the antimicrobial activity of these bacteria (12 potential probiotic bacteria) indicated that *L. plantarum* strain ATCC 8014 exhibited the greatest capacity to inhibit the growth of the three reference strains of *Clostridium*, based on the activity detected using screening methods. In addition to *L. plantarum* ATCC 8014, other species showed inhibitory activity against one or all *Clostridium* strains. However, their antimicrobial activities were evidenced by lower zones of inhibition or were variable in comparison to the inhibition test of gas production. This variability in performance has previously been reported and suggests that more than one method should be used to assess the antimicrobial activity of probiotics, given that the conditions of each methodology may interfere with the results [52]. 

Various species of *Lactobacillus* are able to produce compounds with antimicrobial activities, including organic (acetic and lactic) acids, low-molecular-weight compounds, antifungal peptides, and antibacterial peptides (bacteriocins) [53,54]. It appears, however, that the inhibition of *Clostridium* growth exhibited by the probiotic strains was not the result of the overproduction of acids, since the addition of buffer to the MRS media did not affect the inhibitory activity of the majority of these species. Furthermore, some species of *Clostridium* exhibit intense fermentative activity, resulting in the production of large quantities of organic acids, including acetic, lactic, formic, butyric, and propionic acid, among other substances; thus, they would likely already be habituated to these and would survive acidic pH conditions [54,55]. In addition, as *Lactobacillus* and *Bifidobacterium* can produce a large amount of organic acids [55] and the concentration of the phosphate buffer used in the assay might not be sufficient to neutralize them, we tested the culture supernatants of *L. plantarum* ATCC 8014 at pH 4.3 and 6.5, but the inhibitory effect continued to be evidenced even after pH adjustment, although there was a higher activity against *L. butyricum* at pH 4.3.

This evidence allows us to suggest that other molecules produced by the probiotic strain are expected to be involved in their inhibitory actions on *Clostridium* strains. Indeed, a recent study demonstrated that *Lactobacillus* metabolites isolated from vaginal smears presented with in vitro bacteriostatic effects against *C. perfringens* [56]. These have been associated with the production of bacteriocins, which are produced by several species of probiotics and have bactericidal or bacteriostatic actions. Bacteriocins may increase the permeability of the inner membrane of bacteria, thus contributing to their rupture and interfering with bacterial cell wall synthesis, resulting in pore formation by binding to the peptidoglycan precursor lipid [57]. Lacticin 3147, for example, produced by *Lactococcus lactis*, forms selective pores in the cell walls of some pathogenic gram-positive bacteria, including *C. difficile*, resulting in its death [58].

The nature of the compound(s) produced by *L. plantarum* ATCC 8014 and their potential bactericidal or bacteriostatic actions are not yet known. However, even if such compounds are bacteriostatic or capable of inducing only sporulation, such actions could be relevant to controlling the clinical manifestation of infections caused by *Clostridium*, neutralizing the metabolic activity of the pathogen and, consequently, the production of toxins and other virulence factors involved. In addition, it is worth mentioning that *Clostridium* spp. are gram-positive bacteria and bacteriocins have a more targeted inhibitory action against this type of microorganism rather than against gram-negative bacteria [52,54].

Several criteria are used to define microorganisms as probiotics, including the ability to: (1) coaggregate with microbial pathogens; (2) adhere to eukaryotic cells and mucus; and (3) tolerate conditions of acidic pH and bile salts, among other properties [59,60]. *L. plantarum* ATCC 8014 fulfilled all these criteria, as it tolerated acidic pH and bile salts under the conditions tested, coaggregated with *Clostridium* spp., and exhibited adhesive properties suggesting its capacity for in vivo colonization. Although this bacterium demonstrated lower mucin binding than the *L. fermentum* strain ATCC 23271, the results obtained were of great relevance.

A worrying issue in the selection of probiotics to be used in foods or supplements is their potential to transmit genes involved in antibiotic resistance, especially if the microorganism in question carries plasmids [61,62]. Herein, *L. plantarum* ATCC 8014 showed resistance to ciprofloxacin and vancomycin. However, despite having plasmids [63], resistance to these antibiotics is considered intrinsic and non-transmissible in this species [64,65]. Of importance, vancomycin is among the antibiotic options to treat *Clostridium* infections [66,67]. Thus, we can assume that the development of a therapeutic strategy for intestinal disorders consisting of vancomycin administration in association with *L. plantarum* ATCC 8014 could be more effective than the single use of the antibiotic, if a synergistic effect is to be proven.

Currently, there is a large panel of probiotic strains in use. However, in most cases, probiotic action has been shown to be species-specific or even strain-specific [31,68,69,70]. Thus, the possibility of a potential probiotic with proven antimicrobial action against multiple *Clostridium* species, which are commonly associated with pathologies resulting from an imbalance in the microbiota, represents a significant advance for the reduction of morbidity and mortality rates arising from these clinical syndromes. In addition to being a more rational therapeutic approach, use of a probiotic would not have a negative impact on the intestinal microbiota and would not exert pressure for the selection of resistant bacteria, as with conventional antibiotic therapy [71].

Although the results presented herein are promising in terms of novel therapeutic strategies to treating intestinal infections caused by *Clostridium* spp., especially as *L. plantarum* ATCC 8014 is readily available to the scientific community as a culture collection strain, certain limitations of the study should be taken into consideration. Firstly, although there was an apparent cell monolayer integrity under microscopic examination of HeLa cells, its viability following incubation with the tested probiotic strain was not evaluated; thus, it is uncertain whether the adherence of *L. plantarum* ATCC 8014 was facilitated due to HeLa cell death. Also, it would be important to confirm the probiotic potential of *L. plantarum* ATCC 8014 in assays with clinical isolates of *Clostridium*, as not all reference strains tested in the present study are pathogenic, and to assure whether there is any variability in the spectrum of the antimicrobial activity against a larger panel of pathogenic isolates of *C. butyricum*, *C. difficile*, and *C. perfringens*. It is also important to highlight the need for addressing the safety of *L. plantarum* ATCC 8014 for oral administration in humans, especially in patients who have predisposing factors, such as preterm newborns or critically ill children [40]. Therefore, further studies in animal models and clinical trials would be essential to fill these gaps of knowledge and determine the in vivo benefits of *L. plantarum* ATCC 8014 against *Clostridium* spp.

## 5. Conclusions

Our findings allow us to conclude that *L. plantarum* strain ATCC 8014 has probiotic potential, with antimicrobial activity against *C. butyricum* ATCC 860, *C. difficile* ATCC 9689, and *C. perfringens* ATCC 12924. Additionally, this microorganism fulfills essential criteria to survive the harsh conditions of the gastrointestinal tract, as well as to colonize it.

## Figures and Tables

**Table 1:** Inhibitory activity of *Bifidobacterium* and *Lactobacillus* against *Clostridium* spp. based on the agar spot test.

Potential Probiotics	Diameter of Inhibition Zones (mm ±SD) of:		
	*Clostridium butyricum*	*Clostridium difficile*	*Clostridium perfringens*
*Bifidobacterium animalis* 56.1	0	0	0
*Bifidobacterium bifidum* 14.2	12 (1.6)	12 (0.4)	11 (1.7)
*Bifidobacterium longum* ATCC 15707	11 (0.4)	12 (0.2)	11 (1.4)
*Bifidobacterium longum* 49.3	12 (0.4)	11 (0.0)	10 (0.7)
*Lactobacillus brevis* ATCC 367	9 (0.4)	0	0
*Lactobacillus delbrueckii* ATCC 9649	10 (1.5)	11 (0.7)	12 (2.4)
*Lactobacillus fermentum* ATCC 23271	10 (1.1)	10 (0.5)	10 (0.6)
*Lactobacillus fermentum* 54.2	13 (0.7)	9 (0,4)	10 (0.0)
*Lactobacillus paracasei* ATCC 335	11 (0.5)	12 (0.3)	11 (0.4)
*Lactobacillus plantarum* ATCC 8014	17 (0.8)	13 (1.1)	13 (0.6)
*Lactobacillus rhamnosus* ATCC 9595	11 (0.0)	0	0
*Lactobacillus rhamnosus* GG ATCC 53103	10 (0.9)	0	0

**Table 2:** Inhibition of gas production by *C. butyricum* induced by probiotics grown in MRS medium with and without phosphate buffer.

Strains	Without Buffer	With Buffer
*B. animalis* 56.1	+	+
*B. bifidum* 14.2	+	−
*B. longum* ATCC 15707	+	+
*B. longum* 49.3	−	−
*L. brevis* ATCC 367	+	+
*L. delbrueckii* ATCC 9649	+	+
*L. fermentum* ATCC 23271	−	−
*L. fermentum* 54.2	−	−
*L. paracasei* ATCC 335	+	+
*L. plantarum* ATCC 8014	+	+
*L. rhamnosus* ATCC 9595	+	+

**Table 3:** Antimicrobial activity of cell-free culture supernatants (CFSN) of *L. plantarum* ATCC 8014 at pH 4.3 and 6.5 against *Clostridium* spp. by the agar overlay diffusion.

*Clostridium* spp.	Inhibition Zone Diameters of CFSN in mm (±SD) at:		*t* ^1^	*p* Value ^1^
	pH 4.3	pH 6.5		
*C. butyricum* ATCC 860	16.5 (0.5)	14.7 (0.5)	5.97	0.002
*C. difficile* ATCC 9689	14.2 (0.8)	13.2 (0.4)	2.24	0.076
*C. perfringens* ATCC 12924	14.3 (0.8)	13.7 (0.5)	1.35	0.235

^1^ Comparative analysis was performed by paired Student’s *t*-test (*p* < 0.05).

**Table 4:** Adherence quantification of *L. plantarum* ATCC 8014 to HeLa cells and to mucin in comparison with the control.

Assays ^1^	*L. plantarum* ATCC 8014	*L. fermentum* ATCC 23271	*p* Value ^2^
Cell adhesion	7.602 (±0.135)	7.349 (±0.053)	0.0037
Mucin binding	5.057 (±0.062)	5.370 (±0.031)	<0.0001

^1^ Data are expressed as mean log_10_ CFU/mL (standard deviations) of triplicate experiments performed on three independent days. ^2^ Comparative analysis was performed by Student’s *t*-test (*p* < 0.05).

**Table 5:** Survival of *L. plantarum* ATCC 8014 and *L. fermentum* ATCC 23271 in the presence of acidic pH and bile salts.

Conditions	% Survival (±SD) ^1^		*p* Value ^2^
	*L. plantarum*	*L. fermentum*	
pH 2.0	70.3 (±4.46)	64.7 (±6.49)	0.1155
pH 4.0	97.8 (±5.67)	106.2 (±9.36)	0.7606
Bile salts 0.5%	110.8 (±12.04)	112.7 (±8.79)	0.0912
Bile salts 1.0%	90.1 (±3.77)	92.6 (±3.07)	0.2419

^1^ Data represent survival percentage of microorganisms after 180 min of exposure to distinct conditions in comparison to bacterial growth of each under standard conditions. ^2^ Comparative analysis was performed by the Student’s *t*-test (*p* < 0.05).

**Table 6:** Antimicrobial susceptibility of *L. plantarum* ATCC 8014 by the agar overlay diffusion method.

Antibiotics	Inhibition Zone Diameters mm (±SD)	Interpretation ^1^
Clindamycin	25.7 (1.1)	Susceptible
Chloramphenicol	20.3 (1.5)	
Erythromycin	30.0 (1.0)	
Gentamicin	14.5 (0.5)	
Penicillin	33.7 (1.2)	
Rifampicin	23.3 (0.6)	
Tetracycline	31.0 (1.0)	
Co-trimoxazole	14.3 (0.6)	Moderately susceptible
Ciprofloxacin	9.3 (0.6)	Resistant
Vancomycin	0	

^1^ The interpretive criteria for the diameters of inhibition zones were those described by Chateris et al. [48].
